# Weaving a public health network: Process and results from an evaluation of CDC’s Prevention Research Centers Vaccine Confidence Network^☆^

**DOI:** 10.1016/j.vaccine.2025.127636

**Published:** 2025-08-21

**Authors:** Yulia E. Chuvileva, Nicole M. Kuiper, Aparna Ramakrishnan, Kenneth Goodman, Alyssa Jeffers, Hepsi Swarna, Pooja Gandhi, Melinda Reed-Morrice, Brigette Ulin

**Affiliations:** a Centers for Disease Control and Prevention, National Center for Chronic Disease Prevention and Health Promotion, Division of Adolescent and School Health, 4770 Buford Hwy, Atlanta, GA 30341, USA; b Centers for Disease Control and Prevention, National Center for Chronic Disease Prevention and Health Promotion, Division of Population Health, 4770 Buford Hwy, Atlanta, GA 30341, USA; c Devi Partners, San Carlos, CA 94070, USA; d Karna LLC, 2800 Century Pkwy Ste 700, Atlanta, GA 30345, USA; e Centers for Disease Control and Prevention, National Center for Infectious and Respiratory Diseases, Immunization Services Division, 1600 Clifton Rd. NE, Atlanta, GA 30333, USA

**Keywords:** COVID-19 pandemic, Network evaluation, Collective impact, Social network analysis

## Abstract

As part of its COVID-19 pandemic response, in April 2021, the Centers for Disease Control and Prevention (CDC) funded 26 university-based Prevention Research Centers (PRCs) with a one-year award to raise vaccine confidence, demand, and uptake in diverse communities. The PRCs formed the Vaccine Confidence Network (VCN) to share information, resources, and strategies, and to collaborate. To support the VCN, CDC organized and facilitated numerous network activities, including hosting 14 virtual network meetings, a Slack channel, a resource library, and 4 office hours, disseminating 52 editions of a newsletter, and responding to 172 technical assistance requests from PRCs. These efforts aimed to increase meaningful and impactful collaborations over time so that PRCs’ work benefited from their engagement in the VCN. We evaluated the VCN using two network surveys, an interim one at 6 months and a final one at 16 months, with 26 key informant interviews conducted during interim reporting to guide final survey tool development. The interim survey asked PRCs to report on the levels of collaboration and impact in their PRC-PRC relationships. The final survey repeated the interim survey’s measurement and asked PRCs about the types of collaborations they engaged in with other PRCs, the types of impacts those collaborations had on their work, and how the collaborations came about (their origins). The number of reported undirected connections between the 26 PRCs grew by 72 %, from 53 to 91 between interim and final measurements. The self-reported amounts of collaboration and impact increased over time. The most common type of collaboration was exchange of information, tools, and resources (in 64/91, 70 % of undirected connections). While the most common type of impact was improved design (in 57/118, 48 % of directed connections). Of collaborations whose origins PRCs reported, 86 % (67/78) first connected during a VCN network activity, like chatting in a meeting or in Slack. The VCN example (i.e., its network practices and evaluation) could be useful to funders and self-organizing networks that are looking to enact coordinated responses to emergency and non-emergency public health and other issues.

## 1. Introduction

The response to the 2020–2023 COVID-19 pandemic public health emergency in the United States included the rapid development of safe and effective vaccines, requiring an efficient rollout to protect the population from severe disease and stem the spread of SARS-CoV-2, the virus that causes COVID-19 [1]. However, the vaccine rollout process faced many challenges. Factors such as politicization of vaccination, conspiracy theories and misinformation, inequities in vaccine distribution and access (particularly in early roll-out phases), and ongoing social impacts of past medical injustices experienced by different populations [2–5] led to mistrust of public authorities, medical institutions, and pharmaceutical companies, and reduced public confidence in COVID-19 vaccines [6].

As part of its COVID-19 Response, in April 2021, the US Centers for Disease Control and Prevention (CDC) funded 26 university-based Prevention Research Centers (PRCs) with a one-year award to raise vaccine confidence, demand, and uptake in diverse communities. Since the PRCs already operated as the PRC Network in their usual work, CDC’s COVID-19 Response identified the PRC Network as a potentially impactful way to rapidly raise vaccine confidence, demand, and uptake in groups disproportionately impacted by COVID-19 morbidity and mortality. Additionally, the PRCs had already individually mobilized their existing partnerships and trust with communities to respond to the pandemic by providing needed services in their localities [7]. CDC thus saw a two-fold opportunity with the individual PRCs and the PRC Network: 1) to continue leveraging the centers’ existing expertise and relationships to work with their local communities on vaccine confidence and demand; and, 2) to organize individual PRC COVID-19 vaccine confidence, demand, and uptake efforts into a network to share information, resources, and strategies, to support one another, and to collaborate to advance their individual and collective projects.

Once funded, the PRCs formed the Vaccine Confidence Network (VCN) to leverage collective impact to increase vaccine confidence [8]. Compared with other PRC networks [9–11], the VCN was unique for the PRC Program in several ways: all 26 PRCs engaged in the VCN (rather than a select few), the timeline was extremely limited (one year), and the health topic was new for all PRCs. The award required individual PRCs to coordinate with CDC and health department pandemic response efforts to: assess barriers and facilitators to COVID-19 vaccination in their communities; adapt and implement recommended strategies to increase vaccine confidence, demand, and uptake; and evaluate the effectiveness of their interventions to generate behavioral insights. Collectively, CDC expected the PRCs to participate in the VCN to support each other’s efforts. CDC expected broad geographic reach, diverse populations of focus, and existing relationships among PRCs would result in larger per-community impact in a network than any one center could achieve on its own.

Because no network coordinating center was funded (as is practiced in other CDC-funded PRC networks [9–11]), CDC dedicated its own staff and contractors (including evaluators) to provide VCN coordination and support and take the lead in organizing, facilitating, and evaluating the network. The literature on collective impact uses a term for the role that the CDC played: “the backbone”. This refers to the organization or team that actively and intentionally fosters meaningful connections between network members. We will refer to the organizing CDC group as the “CDC backbone team” throughout this manuscript. In the following, we describe the process the CDC backbone team undertook to rapidly establish and evaluate the VCN and share findings from our network evaluation.

## 2. Materials and methods

### 2.1. Establishing, organizing, and weaving the VCN

To rapidly (and concurrently) assess the PRCs’ programmatic strategies, establish, organize, and weave the VCN (i.e., facilitate meaningful connections), and develop network evaluation data collection tools, we drew extensively upon CDC’s Vaccinate with Confidence framework [12], and academic and gray literature on collective impact [13], impact networks [14], and network weaver [15] approaches.

Strong backbone support is essential to effective collective impact networks. It refers to the labor involved in setting up, running, and facilitating a collective impact network, including by coordinating the work of network members and partners, and managing the collection, analysis, and dissemination of network data [13,14]. To build, organize, and weave the VCN, from June 2021 through September 2022, CDC’s backbone team hosted 14 virtual network meetings, a Slack channel, a shared resource library, and 4 CDC office hours, disseminated 52 editions of the VCN Weekly Roundup newsletter, and responded to 172 technical assistance requests from PRCs.

The virtual network meetings aimed to provide information and support, and to facilitate collaboration among the PRCs. During the network meetings, CDC shared updates on developments in COVID-19 vaccination, provided guidance and resources for developing and evaluating behavioral interventions for immunization, and answered questions regarding the state of the vaccine confidence, demand, and uptake science. PRCs shared updates on their efforts, discussed challenges and how to overcome them, and shared experiences and ideas in breakout groups.

Slack allowed for real-time communication between PRCs to connect and exchange ideas through sub-channels focused on specific strategies, populations, and challenges. The online resource library included tools and templates for PRCs to use, resources developed by PRCs, and files to support coordination. The VCN Weekly Roundup newsletter contained highlights from network meetings, Slack, and the resource library, as well as other relevant updates and collaboration opportunities; it was shared with all VCN members. CDC’s backbone team addressed individual technical assistance requests, which mostly centered on intervention design and evaluation, and connected PRCs with subject matter experts. Office hours provided extra support to PRCs with additional questions or technical assistance needs. The VCN-recommended survey items were developed to allow PRCs to assess vaccine confidence and uptake in their priority communities in a consistent manner across the VCN.

Collective impact and impact network approaches highlight the importance of continuous communication and shared monitoring, measurement, and evaluation in building and maintaining successful inter-organizational networks [13,14]. The VCN was designed to ensure that continuous learning and feedback loops between CDC and PRCs informed the management of network meetings and platforms. For example, ongoing feedback gathered through regular polls guided the choice of topics, activities, and formats of network meetings. The polls measured PRCs’ engagement in calls, intent to connect with others following meetings, and preference for future presentation and breakout topics. The reach of the weekly roundup email was measured by email open and read rates.

The collective impact literature additionally identifies network weaving as an important network practice [15]. Network weaving calls on backbone organizations and teams to actively and intentionally foster meaningful connections between network members. One way this can be done is by connecting members based on shared priorities, goals, and other characteristics they have in common, such as geographic location or populations of focus. For the VCN, the CDC backbone team used PRC project information and network monitoring and evaluation data (more on that below) to identify opportunities to connect PRCs among lines of commonality. Meeting breakout groups, for example, connected PRCs with similar challenges, priority populations, or strategies. The CDC backbone team also strategically implemented a coordinated VCN dissemination strategy, successfully supporting the production of several cross-PRC presentations and manuscripts. Lastly, the CDC team attempted to increase engagement and connections of PRCs that reported fewer collaborations by inviting them to present at network meetings.

### 2.2. Evaluating the VCN

To guide evaluation activities, CDC developed a conceptual model and evaluation plan. Contributions to evaluation planning and implementation came from CDC’s COVID-19 Emergency Response Vaccine Task Force members, vaccination subject matter experts from CDC’s National Center for Immunization and Respiratory Diseases (NCIRD), leadership from the Division of Population Health (DPH) of CDC’s National Center for Chronic Disease Prevention and Health Promotion (NCCDPHP), where the PRC Program resided^1^, and the VCN evaluation workgroup made up of PRC representatives. The evaluation plan and questions were organized into three evaluation domains:

- Evaluation Domain 1: Evaluating the processes and outcomes of rapidly building, organizing, and weaving PRCs into a cohesive network.
- Evaluation Domain 2: Monitoring individual PRCs’ progress.
- Evaluation Domain 3: Evaluating individual PRCs’ project outcomes.

We report only on the portion of Evaluation Domain 1 that explores whether, how, how much, and to what effect the PRCs collaborated in their work and whether those collaborations originated from VCN backbone team’s activities. Our mixed methods evaluation used network surveys and interviews to understand:

- Whether the number of collaborative relationships in the overall network (number of connections in the network) and the number of connections for each PRC (degree centrality) increased over time.
- Whether PRCs’ perceived levels of collaboration with other PRCs increased over time (amount of collaboration), and how they collaborated with each other (types of collaboration).
- Whether the level of impact from the collaborations (amount of impact) increased over time, and how the collaborations impacted PRCs’ programmatic work (types of impact).
- How the VCN collaborations began (origins of collaboration) and the role that the CDC backbone team’s supports, prior PRC-PRC relationships, and other factors played in collaboration formation.

### 2.3. Data collection

We requested information on PRC-to-PRC connections in a network survey and interviews 6 months into the one-year award (interim measurement in November 2021) and in an additional network survey 90 days after work ended (final measurement in September 2022). We used interim evaluation findings for individual program improvements, to facilitate collaborations between VCN members (i.e., do network weaving), and to guide the development of the final network survey instrument.

We administered the VCN network surveys using an Excel form (Microsoft Corporation) and held virtual interviews via Zoom (Zoom Video Communications). Individual participants were assured of anonymity and provided verbal informed consent at the start of the interviews. The evaluation team kept no record of which PRC staff filled out the evaluation forms and participated in interviews, nor did we collect any personally identifiable information from respondents.

The VCN evaluation plan was reviewed by CDC, deemed research not involving human subjects, and conducted consistent with applicable federal laws and CDC policies.

#### 2.3.1. Interim network survey

The interim network survey asked each PRC to indicate their level of collaboration with each of the other PRCs (“a little,” _“_a moderate amount,” or “a lot,” or left blank if no collaboration) and the level of impact of that collaboration on their projects (“a little,” “a moderate amount,” or “a lot,” or left blank if no impact yet). PRC staff were instructed to fill out the survey as a team to ensure accuracy and completeness of the information provided.

#### 2.3.2. Interim interviews

We conducted 15-to-20-min semi-structured key informant interviews on the types, origins, and impacts of cross-PRC collaborations in the VCN with members of each PRC team after they submitted their interim network survey responses. All PRC team members working on the award were invited to attend the interviews; 3–5 PRC staff members participated in each interview. CDC interviewers discussed with each PRC team the collaborations reported in their surveys, detailing how they collaborated and what kinds of impacts those collaborations had on their VCN work. The interviews aimed to distill key constructs for types of collaboration and impacts to be measured systematically using the final survey.

Notetakers recorded and transcribed each interview, and two team members analyzed the transcripts using NVivo release 1.7 (Lumivero), inductively coding for the types of collaborations and types of collaboration impacts. The team met frequently to refine the codebook and ensure consistent use of codes. We used the qualitative results to create multi-select dropdown answer options to new network questions included in the final VCN network survey about the types of VCN collaborations and impacts PRCs experienced.

#### 2.3.3. Final network survey

**Fig. 1.**
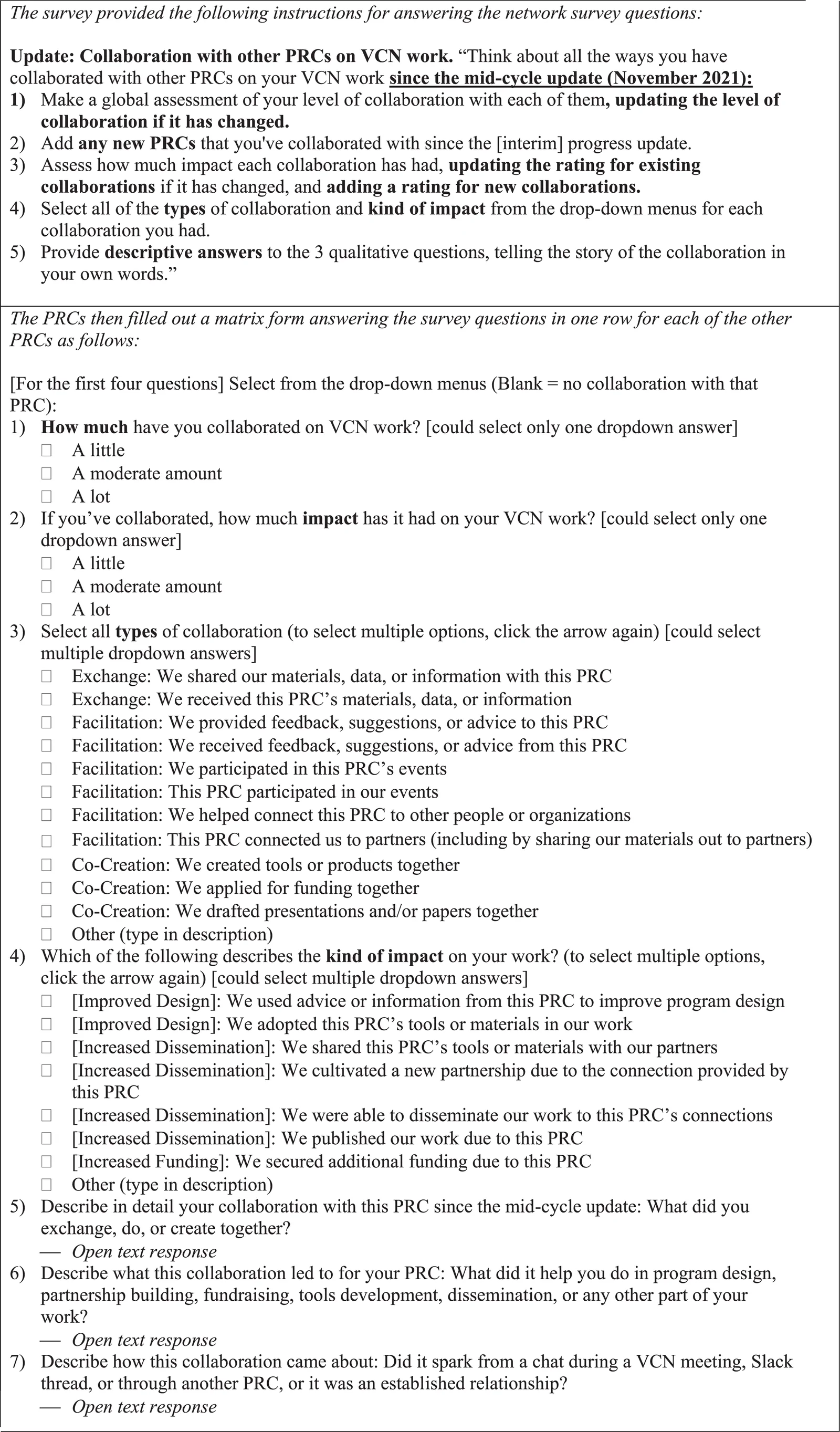
Final network survey instructions, questions, and answer options.

The final network survey provided PRCs with the amount of collaboration and impact data they submitted at interim measurement and asked them to indicate whether anything had changed. Respondents were asked to select all relevant options from dropdown lists for sub-types of collaboration and impact and to describe in detail what their collaborations entailed, what (if anything) they led to, and how they came about. Fig. 1 details the final survey instructions, questions, and answer options.

#### 2.3.4. Quality control measures

CDC implemented quality control measures once PRCs submitted their responses to the interim and final network surveys to resolve incomplete responses and revise information that was provided during interim interviews but not reflected in survey responses. The response rate for the network survey at both time points was 100 %. We extracted all qualitative and quantitative data from the surveys into Excel and uploaded Excel network data into Kumu (Kumu.io version 3.3) for network visualization and calculation of degree centrality. Data were stored in and managed by the evaluation team through a secure CDC share filing system and are not available publicly.

### 2.4. Data analysis and visualization

#### 2.4.1. Qualitative data analysis

We analyzed qualitative descriptions of collaboration types, impacts, and origins using a two-rater coding system. The first rater analyzed the available qualitative information for each reported PRC-to-PRC collaboration to assess the origins of the collaboration, the sub-types of collaboration, and the sub-types of impact reported according to the constructs developed from the interim interviews. The second rater then repeated the analysis, indicating whether they agreed or disagreed with the first rater’s codes. All differences were resolved through discussion to come to a consensus. The raters refined and updated the codebook in the process, recoding items if changes were made. This process led to the addition of an increased dissemination impact sub-type of “We are currently discussing publishing together or are actively working towards a publication together” to the data analysis, which was not included in the final network survey form, but which was prevalent in PRCs’ qualitative descriptions of impacts they derived from their VCN collaborations. The raters’ final agreed-upon codes were compared and combined with the codes reported by the PRCs in the closed answer portion of the final network survey, so that if the raters agreed on a code from the survey narratives that the PRCs did not select from the dropdown options, that code was added to that PRC’s results.

#### 2.4.2. Network data analysis and visualization.

To explore VCN’s network structure and the changing relationships between PRCs, we visualized the data into network maps using Kumu, calculating degree centrality (number of connections each PRC was reported to have with other PRCs) in the platform. Since networks consist of dependent observations, traditional statistical testing is not appropriate [16]. Specialized statistical tests for longitudinal, closed network data (such as stochastic actor-oriented models [17]) produce complex predictive models that are beyond the scope of the present descriptive study. Consistent with previous research exploring changes in organizational collaboratives, we compare descriptives across the two time points to ascertain general trends in the data without generating *p* values [18].

In social network analysis, relationships can be either directed or undirected. Directed ties represent asymmetric relationships—such as one-way information sharing or funding—where reciprocity is not assumed. Undirected ties, by contrast, imply mutual engagement, such as joint discussions or co-creation efforts. Accordingly, directed graphs can contain up to twice the number of ties as undirected graphs, as each dyad can include two directional ties (A → B and B → A).

We report our core visualization and baseline network statistics, i.e., number of connections (number of dyads of PRC-PRC relationships) in the VCN and degree centrality for each PRC, at interim and final measurements, using symmetrized (undirected) data. Symmetrization was applied such that a collaboration tie was considered present if either PRC in the dyad reported the relationship. Symmetrization was appropriate given the collaborative context and the theoretical expectation of mutual engagement in each collaboration. Applying the weak symmetrization rule (as opposed to the strong rule where a tie would only be counted if both PRCs in a dyad reported the relationship) was appropriate given the expectation of under-reporting due to institutional memory loss (see limitations section).

Although we treat the presence of PRC-PRC collaborative relationships as conceptually reciprocal, self-reported assessments of collaboration amounts (collaborated “a little,” “a moderate amount,” or “a lot”) were subjective and thus varied across PRCs. For example, one PRC might describe a tie as involving “a little” collaboration, while its counterpart might describe the same relationship as involving “a moderate amount” of collaboration based on their differing baselines of overall network activity. To capture this nuance and preserve respondent-specific variation, we treated reported amount of collaboration as a directed variable.

We analyze the reported amount of impact from VCN collaborations as a directed variable for two reasons. Each PRC assessed the impact of the collaboration from its own perspective, without assuming reciprocal benefit. As such, the directionality reflects a unidirectional flow of perceived impact (e.g., PRC A reports receiving a certain amount of benefit from its collaboration with PRC B). In addition, as with amount of collaboration, amount of impact is subjective and relative to each PRC’s overall impact derived from VCN collaborations.

To accurately reflect the nature of the types of collaborations, impacts, and origins data, we treated primary and sub-types according to whether they were conceptually and empirically directed or undirected:

- *Primary types of collaboration and impact:* The evaluation identified three primary types of collaboration (exchange, facilitation, and co-creation) and four primary types of impact (improved design, increased dissemination, expanded partnerships, and increased funding). PRCs did not report on these primary types directly. Rather, they reported the presence of sub-types of collaboration and impact. To arrive at estimates of the presence of primary types of collaboration and impact in each dyad, and the co-occurrence of primary types of collaboration, we symmetrized the data using the weak rule and thus report it as a proportion of reported undirected VCN relationships. For instance, a primary collaboration type tie (e. g., exchange) was considered present if either PRC in a dyad reported that a sub-type of that primary collaboration type existed in the relationship (e.g., either PRC said one or more of the following exchange sub-types was present: “We shared our materials, data, or information with this PRC” or “We received this PRC’s materials, data, or information”).
- *Sub-types of collaboration:* Primary collaboration types of exchange and facilitation included directed sub-types (e.g., exchange has two directed sub-types “We shared our data with this PRC” and “We received data from this PRC”). As a result, we do not symmetrize these data but report sub-type presence as a proportion of all reported directed connections in the VCN. However, the primary collaboration type of co-creation includes undirected sub-types (e.g., both PRCs in a dyad participate in all three sub-types of “We drafted presentations and/or papers together,” “We are currently discussing publishing together or are actively working towards a publication together,” and “We created tools or products together”). We thus symmetrized co-creation sub-type data using the weak symmetrization rule and report sub-type presence as a proportion of undirected ties in the VCN.
- *Sub-types of impact:* All impact sub-types (across the four primary impact types) are directional, so their data are not symmetrized and reported as a proportion of directed VCN connections.
- *Collaboration origins:* This attribute reflects the context or facilitation of the tie’s formation (e.g., whether the CDC VCN team helped initiate the connection) and does not imply directionality between PRCs. Collaboration origins data were thus symmetrized using the weak symmetrization rule, so that a primary origin type (e.g., VCN) or sub-type (e.g. VCN meeting) was deemed present in a dyad if either PRC reported it as an origin of the collaboration (i.e., we report it as an undirected tie).

## 3. Results

We report the results of the VCN network evaluation according to reporting guidelines for social networks in health research [19].

### 3.1. Network connections and PRCs’ degree centrality

**Fig. 2.**
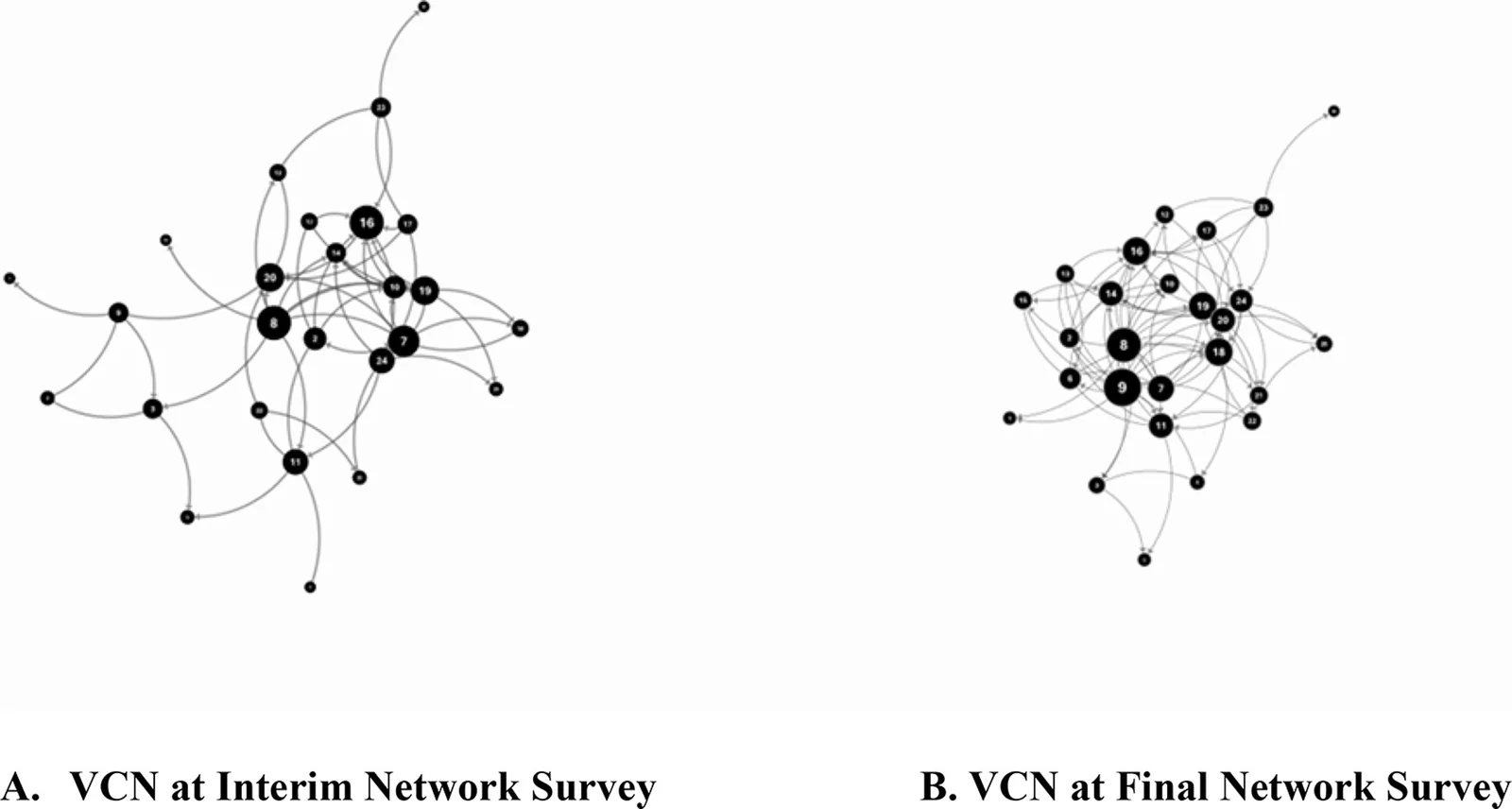
Total number of connections and variation in undirected degree centralities (i.e., differences in the size of elements) in the vaccine confidence network at interim and final reporting.

The number of undirected connections in the whole VCN network the PRCs reported grew by 72 %, from 53 at interim measurement to 91 at final measurement. Fig. 2 visualizes this change. The circles on the map (i.e., the elements) are the individual PRCs and the lines are the reported connections between them. The maps are generated to a consistent scale, so the tighter grouping of elements and connections in graph B compared with graph A illustrates the increase in the number of connections, pulling the overall network into a more tightly woven structure.

In the interim network survey, PRCs’ degree centrality (i.e., the number of all reported undirected connections between the given PRC and other PRCs in the network) ranged from 1 to 9 per PRC, with a mean of 4.1 and a median of 4.0. In the final network survey, the degree centrality ranged from 1 to 17 per PRC, with a mean of 7.0 and a median of 6.0. The mean and median degree centrality increased over time by 72 % and 50 %, respectively, while no PRCs were unconnected to the rest of the network at either time points. Fig. 2 varies the size of each PRC circle according to its degree centrality, showing PRCs with higher degree centrality to be generally more centrally positioned in the network and PRCs with lower degree centrality positioned towards the margins of the network.

### 3.2. Amount and type of collaboration

The percentage of PRC-PRC connections in the directed network that was reported to collaborate “a little” decreased from 94 % (66/70) of connections at interim to 87 % (103/118) of connections at final measurement. The percentage of directed connections in the VCN that was reported to collaborate “a moderate amount” increased from 6 % (4/70) at interim to 8 % (9/118) at final measurement. At interim measurement no PRC reported collaborating with another PRC “a lot;” in contrast, by final measurement, 5 % (6/118) of directed PRC-PRC connections were reported to collaborate “a lot.”

Any given PRC-PRC connection (each dyad) could have multiple ways in which PRCs collaborated (types of collaboration), including changes over time when they might have, for example, started out exchanging information and progressed towards co-developing a manuscript. Overall, 70 % (64/91) of connections in the *undirected* VCN network involved exchange, 38 % (35/91) involved facilitation, and 35 % (32/91) involved co-creation. A little less than half (46 %, 42/91) of the connections in the *undirected* network involved more than one type of collaboration. Most frequent overlaps were between exchange and facilitation (32 %, 29/91 of connections) and exchange and co-creation (19 %, 17/91), while 7 % (6/91) of connections reported both facilitation and co-creation activities, and 5 % (5/91) of connections reported all three types of collaboration activities.

Table 1 shows the frequency of collaboration sub-types reported for the VCN network at final measurement only. For exchange, PRCs more frequently reported sharing their materials, data, or information with other PRCs (in 49 %, 58/118 of *directed* VCN connections) than receiving them (in 39 %, 46/118 of *directed* VCN connections). The most frequent sub-types of facilitation collaborations were providing feedback, suggestions, or advice to other PRCs (in 15 %, 18/118 of *directed* VCN connections) and receiving the same from other PRCs (in 11 %, 13/118 of *directed* VCN connections). The most frequent forms of co-creation PRCs engaged in were drafting presentations or papers together (in 22 %, 20/91 of *undirected* VCN connections) and discussing collaborating on manuscripts (in 11 %, 10/91 of *undirected* VCN connections).

### 3.3. Amount and type of impact

The amount of impact reported at the two evaluation time points followed a similar trend as the amount of collaboration. Although the percentage of *directed* connections with “a little” impact decreased from 90 % (63/70) of connections in the VCN at interim measurement to 81 % (96/118) at final measurement, the percentage of connections that had “a moderate amount” of impact increased from 10 % (7/70) to 15 % (18/118) across the two time points. While at interim measurement, no PRC reported “a lot” of impact from their collaboration with another PRC, by final measurement, 4 % (5/118) of the *directed* connections were reported to have had “a lot” of impact on PRCs’ VCN work.

Across VCN’s *directed* network, the most reported type of impact was improved design in 48 % (57/118) connections, with 96 % (25/26) of PRCs reporting improving the design of their projects because of their VCN collaborations. Second most common reported impact was increased dissemination (43 %, 51/118), followed by expanded partnerships (18 %, 10/118). No PRC reported securing increased funding because of their VCN relationships with other PRCs.

Table 2 details the frequency of each reported sub-type of impact as a percentage of all *directed* VCN connections. For improved design, PRCs most frequently reported using advice or information from other PRCs to improve program design (36 %, 43/118 of *directed* connections) and least frequently adopting another PRC’s tools or materials in their own work (18 %, 10/118 of *directed* connections). Meanwhile, the most frequently reported sub-type of increased dissemination was currently discussing manuscript co-production (22 %, 26/118). Some of these dissemination collaborations led to the collaborative manuscripts contained in this *Vaccine* journal supplement. Overall, 73 % (19/26) of PRCs reported dissemination as an impact of at least one of their collaborative relationships with other PRCs.

### 3.4. Origins of PRC-PRC connections

**Fig. 3.**
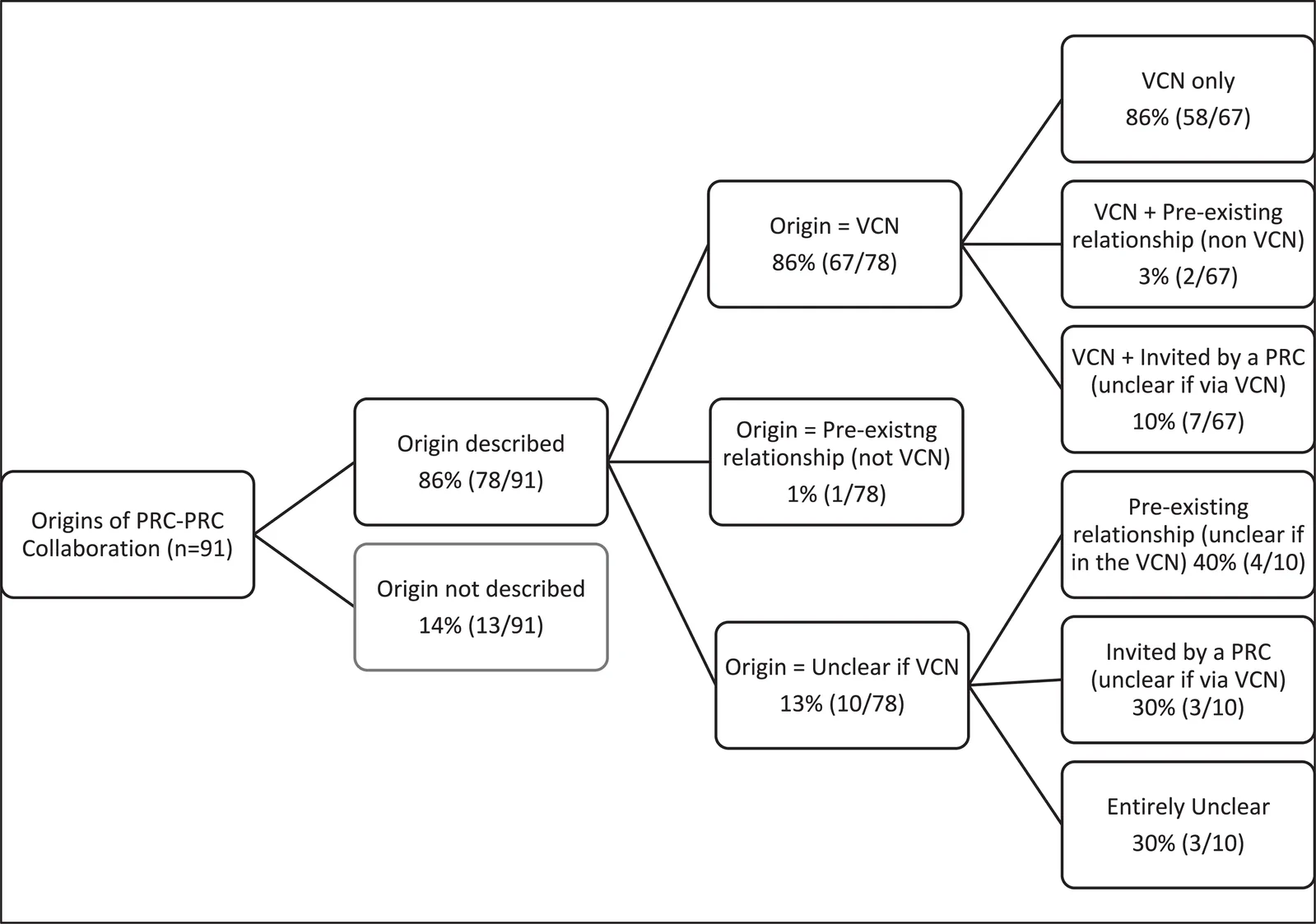
Tree diagram of reported origins of undirected PRC-PRC collaborations.

The tree diagram in Fig. 3 visualizes the data for the origins of *undirected* PRC-PRC VCN connections. In the final network survey, PRCs provided written descriptions of the origins of their PRC-PRC collaborations for 86 % (78/91) of *undirected* connections. For the remaining 14 % (13/91) of *undirected* connections, the origins were not described. Among the 78 connections with origin descriptions, 86 % (67/78) clearly indicated that the collaboration was established through the VCN meetings and communication channels organized and facilitated by the CDC backbone team (i.e., CDC’s network weaving activities). Of the remaining 11 *undirected* connections, 1 % (1/78) was explicitly stated to not have been due to the VCN but had originated from a pre-existing relationship between the two PRCs. For 13 % (10/78) of *undirected* connections, it was not clear from the descriptions whether the relationship came out of a VCN connection point. Of the unclear descriptions: 40 % (4/10) mentioned an existing relationship but it was not clear whether the relationship pre-existed in the VCN or outside the VCN; 30 % (3/10) mentioned being invited by another PRC into the collaboration but did not explicitly say whether that was through the VCN; and the rest (30 %, 7/10) were completely unclear.

Among the *undirected* connections for which PRCs clearly described a VCN origin, 86 % (58/67) mentioned connecting only through VCN touch points (i.e., not through other avenues). A couple (3 %, 2/67) mentioned also having a pre-existing relationship with a PRC. For instance, one PRC noted their collaboration with another PRC, stating, “Originally, [we collaborated] from an ongoing partnership with [this PRC], but also through dissemination discussions in the VCN meetings.” And 10 % (7/67) additionally mentioned being invited by a PRC into a collaboration but it was not clear whether that invitation happened through the VCN.

**Fig. 4.**
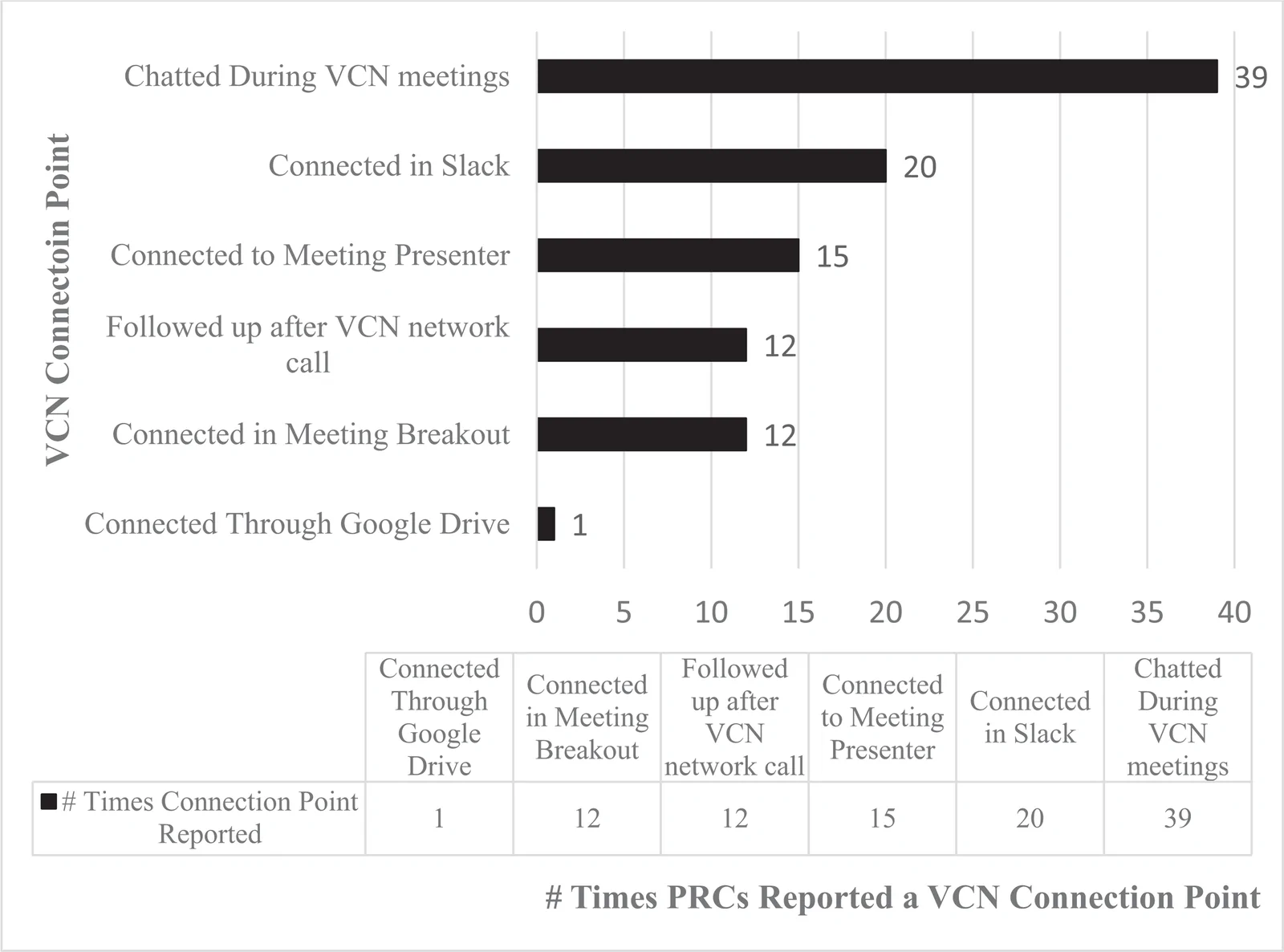
Frequency of connection touch points reported in the Vaccine Confidence Network.

Finally, of the *undirected* connections with a clear VCN origin, two thirds (64 %, 43/67) reported connecting through a single VCN connection point, such as the Slack channel or in a meeting breakout, while one third (36 %, 24/67) naming more than one VCN connection touch point as the origin of their collaboration, sometimes sequentially. For example, first chatting in a meeting then connecting in a conversation in a breakout room of the same session, and then attending a post-VCN-meeting follow up call. Fig. 4 shows the most frequently used VCN connection touch points. Using the chat function during VCN meetings was the most frequently used connection method (in 57 %, 38/67 of *undirected* VCN-originated connections), followed by the Slack channel (in 30 %, 20/67 of VCN-originated connections), and talking with a meeting presenter in a session (in 21 %, 14/67 of VCN-originated connections).

## 4. Discussion

Our analysis shows the growth of the VCN network over the course of the one-year award, demonstrating that the VCN achieved its goals of increasing connectivity and impact over time. The total number of connections and the mean and median of individual PRCs’ degree centrality increased between the interim and final measurement points. Similarly, the amount of collaboration and impact reported by the PRCs also increased.

Throughout the VCN, PRCs engaged in exchange, facilitation, and co-creation to varying degrees, with almost half of the PRC-PRC connections collaborating in multiple ways. PRCs reported various impacts of these collaborations, including improving the design of their vaccine confidence programs, increasing the dissemination of their work, and expanding their partnerships. For example, the finding that exchange was the most frequent type of collaboration demonstrates the importance of sharing information and resources in a network like VCN. This kind of exchange allowed PRCs to increase available resources and apply what they learned to improve the design of their projects.

The fact that all but one of the 26 PRCs reported improving the design of their vaccine confidence, demand, and uptake projects in about half of all VCN connections aligns with PRC staff perceptions that VCN directly impacted their work with their priority communities. PRC leaders and staff perceived that their COVID-19 work was enhanced because of their participation in the VCN. The finding that 19 out of 26 PRCs reported increasing the dissemination of their work (by actions like planning to and publishing manuscripts together or distributing other PRCs’ work to their own networks) implies that both the PRCs and the vaccine confidence field at large likely benefitted from the PRCs’ participation in the CDC-funded network.

PRCs reported that the vast majority (86 %) of their connections with other PRCs originated from the network facilitation and weaving work conducted by the CDC backbone team. This finding reflects existing evidence of the importance of backbone organizing labor in collective impact networks [11,12]. Two-thirds of the VCN-originated connections were established through a single point of VCN origin (such as connecting in a network meeting or through a Slack channel). Conversely, a third of VCN-originated connections were reported to have come about through more than one VCN touchpoint, indicating that providing multiple opportunities to connect may be important to network building. That 9 % of connections additionally involved a prior PRC-PRC relationship lends some evidence to CDC’s initial assumption that PRCs would be able to leverage their existing relationships in the PRC Network to advance their vaccine confidence, demand, and uptake projects. This percentage was not higher likely because PRCs hired many new staff to run their VCN projects, staff who were not in the PRC Network previously and thus did not have personal prior connections, even if their institutions did. In the case of VCN, it was the CDC backbone team’s network building, organizing, and weaving work (through hosting meetings, organizing strategic breakout groups, providing various communications channels, etc.) that was the most reported factor in developing PRC-PRC collaborations.

### 4.1. Limitations

Although desirability bias may have led some PRCs to report more connections than existed, we suspect this was minimal since the CDC backbone team was deeply embedded in the VCN program and broadly aware of many of the collaborations among PRCs. The backbone team checked the data we received, including following up with PRCs where clarification about their reported connections was needed. However, since expected types of collaboration were only shared during final reporting, this may have biased the results to indicate a larger increase in collaborations than occurred.

Under-reporting may have been a bigger problem in the dataset. Over half the symmetricized (*undirected*) connections were reported by only one PRC in the dyad, rather than both PRCs (53 %, 28/53 at interim and 58 %, 53/91 at final measurement). Ideally, each connection would have been reported by both PRCs in each dyad. Recall issues might have led some PRCs to under-report their connections with others, especially since each PRC team working on VCN included multiple people whose memory would have needed to be tapped to collectively answer the network surveys as a group. Like many organizations, PRCs struggled with staff turnover, especially under the chaotic circumstances of the COVID-19 pandemic, which may have contributed to any loss of institutional memory of connection in the VCN.

Another reason for under-reporting of connections may have been varying interpretations of collaboration. During interim reporting, we received feedback that a few PRCs may have only reported significant relationships that involved co-creation, leaving out relationships of exchange and facilitation. In response, we corrected this reporting at interim where we came across it, ensured that clear definitions were shared during final reporting, and provided PRCs with additional technical assistance in completing the final network survey in an all-hands VCN meeting where we discussed survey definitions and logistics and answeresd PRCs’ questions. If under-reporting was higher at interim than at final measurement, the increases in connections for the whole network reported in this manuscript may be over-estimated.

Another limitation of the study is that we asked PRCs to subjectively report collaboration and impact amounts using a scale of “a little,” “a moderate amount,” or “a lot.” It is likely that each PRC had its own slightly varied and relative definition of what “a lot” versus “a little” looked like. However, these were just two measures in the network survey that show an increase in amount of collaboration and impact over time, while the other more objective qualitative measures of the types of connection and impacts PRCs reported round out the VCN network evaluation.

## 5. Conclusion

The VCN evaluation confirms that a temporary network of academic research centers can successfully be rapidly organized and implemented to collaborate on work to address acute public health emergencies. Such a network structure benefits from dedicated logistical resources and subject matter and evaluation expertise to set up, facilitate, and weave the network. Thoughtful, intentional backbone work, especially the facilitation of meaningful connections between network members, can help increase the number, amount, type, and impact of collaborations over time. As a result, the work of network members may be enhanced, possibly contributing to greater impact collectively than each individual center could achieve on their own. In other words, facilitating network collaboration could result in increased individual and collective impact. The VCN example (i.e., the practices the CDC backbone team engaged in to establish, weave, and evaluate the network) offers a useful model for funders aiming to support coordinated responses to public health and other challenges and for self-organizing networks seeking collective impact.

## Figures and Tables

**Table 1:** Frequency of collaboration sub-types reported for the vaccine confidence network.

Primary Collaboration Type	Collaboration Sub-Type	#/% directed or undirected connections
Exchange	We shared our materials, data, or information with this PRC We received this PRC’s materials, data, or information	58/118 (49 %) 46/118 (39 %)
Facilitation	We provided feedback, suggestions, or advice to this PRC We received feedback, suggestions, or advice from this PRC We helped connect this PRC to other people or organizations	18/118 (15 %) 13/118 (11 %) 6/118 (5 %)
	We participated in this PRC’s events	4/118 (3 %)
	This PRC connected us to partners (including by sharing our materials out with partners)	3/118 (3 %)
	This PRC participated in our events	2/118 (2 %)
Co-creation	We drafted presentations and/or papers together We are currently discussing publishing together or are actively	20/91 (22 %)
	working towards a publication together	10/91 (11 %)
	We created tools or products together	2/91 (2 %)

**Table 2:** Frequency of impact sub-types reported for the vaccine confidence network.

Primary Impact Type	Impact Sub-Type	#/% directed connections
Improved Design	We used advice or information from this PRC to improve program design Not specified	43/118 (36 %) 25/118 (21 %)
	We adopted this PRC’s tools or materials in our work	10/118 (8 %)
Increased Dissemination	TBD: We are currently discussing publishing together or are actively working towards a publication together Completed: We shared this PRC’s tools or materials with our partners Completed: We were able to disseminate our work to this PRC’s connections	26/118 (22 %) 14/118 (12 %) 13/118 (11 %)
	Completed: We published our work due to this PRC	4/118 (3 %)
	Completed: Not specified	2/118 (2 %)
Expanded Partnerships	We cultivated a new partnership due to the connection provided by this PRC	10/118 (8 %)
Increased Funding	We secured additional funding due to this PRC.	0/118 (0 %)

## Data Availability

Data will be made available on request.

## Footnotes

1 All staff in CDC’s Division of Population Health, including program, evaluation, and communication staff administering and supporting the PRCs, were subjected to a Reduction in Force in April 2025 that has left the PRC Program’s future in doubt.
